# Cold-Chain Compatible Ethyl Formate Fumigation for Phytosanitary Disinfestation of *Drosophila suzukii* in Blueberries

**DOI:** 10.3390/insects17060580

**Published:** 2026-06-02

**Authors:** Changyao Shan, Li Li, Hang Zou, Ronghua Chen, Baishu Li, Tao Liu

**Affiliations:** 1 Institute of Equipment Technology, Chinese Academy of Quality and Inspection & Testing, No. A3, Gaobeidianbeilu, Chaoyang District, Beijing 100123, China; 34424808@student.murdoch.edu.au (C.S.); lili@caiq.org.cn (L.L.); 25a0904188@cjlu.edu.cn (H.Z.); sy20243193596@cau.edu.cn (R.C.); 2 Harry Butler Institute, Murdoch University, Murdoch, WA 6150, Australia; 3 College of Life Sciences, China Jiliang University, Hangzhou 310018, China; 4 Department of Plant Biosecurity, College of Plant Protection, China Agricultural University, Beijing 100193, China

**Keywords:** methyl bromide alternative, postharvest treatment, cold-chain processing, *Drosophila suzukii*, blueberry quality

## Abstract

Ethyl formate (EF) is a promising alternative to methyl bromide for postharvest disinfestation, but berry export chains require efficacy under refrigerated handling without loss of marketable quality. Using spotted-wing drosophila (*Drosophila suzukii*) in blueberries as a cold-chain model, we compared stage-specific tolerance at 5 to 15 °C and identified 1-day-old eggs as the most tolerant stage, with mortality strongly temperature dependent. Egg-stage concentration to mortality relationships were quantified under 4 h exposures at 5, 10, and 15 °C to nominate temperature-specific intensities within EF flammability safety limits. Candidate schedules (69, 83, and 94 mg·L^−1^ for 4 h at 5, 10, and 15 °C) were confirmed by verification-scale zero-survivor tests under simulated cold-chain conditions, achieving complete control of the most tolerant egg stage. Post-treatment quality was assessed after 1, 7, and 14 days of cold storage. Weight loss, firmness, total soluble solids, titratable acidity, sucrose, and proanthocyanidins were driven mainly by storage time and temperature. EF effects were limited to transiently elevated respiration early in storage, with no detectable injury, decay, or adverse changes in appearance or other physicochemical attributes. Overall, EF provides a cold-chain compatible processing window integrating efficacy, operational safety, and quality preservation for refrigerated blueberry export logistics.

## Simple Summary

Blueberries are highly valued fresh fruit that are often transported under refrigerated conditions, but they can be infested by spotted-wing drosophila, an insect pest that lays eggs inside healthy fruit. This creates a challenge for export because treatments must provide reliable pest control without reducing fruit quality. In this study, we evaluated ethyl formate as a cold-chain compatible treatment for blueberries. We compared its effectiveness at different low temperatures and then confirmed suitable treatment conditions in larger-scale trials. We found that eggs were the most tolerant stage, and the treatment level required for control increased as temperature increased. The selected treatments achieved complete control in large-scale tests. Importantly, treated fruit maintained acceptable appearance and showed no clear long-term decline in quality during storage. These results indicate that ethyl formate has potential as a practical postharvest treatment for blueberries, helping support export trade while reducing the risk of spreading this important pest.

## 1. Introduction

International trade in fresh berries has expanded rapidly under modern cold-chain logistics, yet the same speed and scale that enable premium markets also amplify phytosanitary risk [1]. For high-value commodities such as blueberries, quarantine interceptions can be disproportionately costly, leading to shipment delays, consignment rejection, intensified inspection frequency, and, in some contexts, temporary suspension of market access. In parallel, regulators and industry face increasing pressure to adopt postharvest disinfestation options that are operationally scalable and environmentally acceptable, rather than relying on legacy fumigants with tightening restrictions or on treatment systems that are difficult to standardize and audit at scale [2]. Consequently, the field is shifting from one-off laboratory demonstrations toward treatment frameworks that can be implemented, monitored, and verified under commercial handling conditions, supported by objective indicators that translate across facilities and seasons [3,4,5].

*Drosophila suzukii* (Matsumura) (Diptera: Drosophilidae), also known as spotted-wing drosophila, is especially problematic for berry industries because females can oviposit into healthy, ripening, intact fruit using a heavily sclerotised, serrated ovipositor that penetrates the fruit skin [6,7]. Unlike most drosophilids that typically exploit damaged, overripe, or fermenting fruit, this behaviour enables infestation of otherwise marketable berries before harvest [7]. Because immature stages develop internally, infestations may show few external symptoms during packing and inspection, reducing the effectiveness of visual sorting and increasing the likelihood of inadvertent transboundary movement in commercially acceptable fruit [8]. Detections therefore often occur late in distribution chains or at border points, when mitigation options are limited and the economic consequences of rejection or market disruption are greatest. This challenge is amplified under cold-chain handling because low temperatures that preserve berry quality also depress insect metabolism, potentially reducing the speed or reliability of rapid postharvest interventions and motivating treatments that remain efficacious under commercially realistic refrigerated conditions [9,10].

Postharvest disinfestation options include cold disinfestation, heat, irradiation, controlled atmospheres, and fumigation [11]. Feasibility is shaped by throughput, commodity tolerance, and defensible compliance records. Cold disinfestation requires continuous attainment and documentation of approved time to temperature schedules, yet commercial cold chains often show within-load temperature heterogeneity and unavoidable fluctuations, so refrigeration may slow pest development but rarely provides stand-alone quarantine assurance unless compliance can be demonstrated [12]. Heat and irradiation can provide quarantine control for some pest to commodity systems, but scale-up is constrained by challenges in uniform treatment delivery and potential quality penalties for heat-sensitive produce, as well as by facility availability, regulatory settings, and market acceptance for irradiation [13]. Fumigation therefore remains widely used because treatments can be completed within hours, yet reliance on legacy fumigants faces tightening constraints associated with environmental regulation and commodity injury risk, reinforcing the need for scalable, lower-impact alternatives compatible with commercial handling [14,15]. These constraints are most acute for berries, where market value depends on appearance and texture and deterioration accelerates with any loss of time to temperature control.

**Figure 1:**
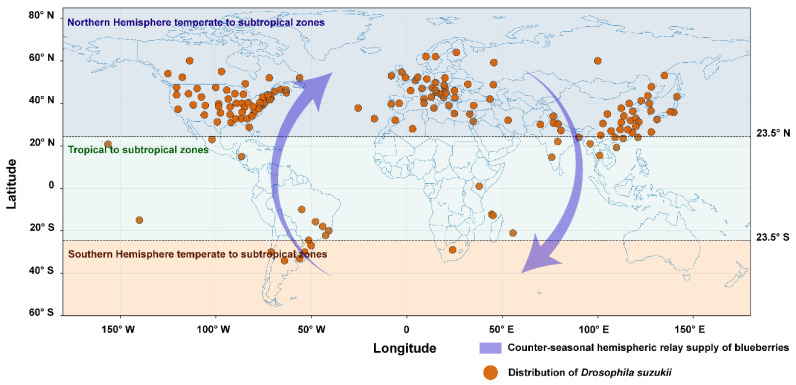
Global correspondence between cross-hemispheric, counter-seasonal blueberry supply and the distribution of *Drosophila suzukii* (Data source: CABI).

Blueberries are a commercially important berry commodity in international fresh-fruit trade, supported by strong consumer demand, premium unit value, and long-distance export pathways that increasingly rely on refrigerated logistics, making them a relevant model for evaluating disinfestation interventions that must protect market access while preserving marketable quality [1,16]. Because blueberry supply is often counter-seasonal and cross-regional, traded pathways may overlap with temperate and subtropical fruit-production regions where *D*. *suzukii* is established, as illustrated in Figure 1. This overlap reinforces the need for cold-chain compatible phytosanitary treatments that provide reliable disinfestation without compromising fruit quality. Blueberries are highly perishable, prone to water loss and decay, and maximum market life depends on rapid removal of field heat and early entry into near-zero cold storage under high relative humidity [17]. Although blueberries are widely regarded as non-climacteric, cultivar-dependent ethylene-related behaviour and atypical ripening features have been reported [18]. Regardless of classification, postharvest performance is dominated by preservation rather than improvement, so handling delays and temperature elevation accelerate softening and decay development [17,19]. Accordingly, interventions that require warming above refrigerated setpoints, including some legacy fumigation practices such as methyl bromide, can disrupt cold-chain protection and increase the risk of quality loss [20,21]. This motivates evaluation of alternative strategies that operate within refrigerated handling while maintaining both quarantine performance and marketable quality throughout distribution [19].

Ethyl formate (EF) has attracted increasing attention as a candidate alternative fumigant because it acts rapidly and has been evaluated across multiple commodity to pest systems in quarantine and postharvest contexts [9]. However, EF performance is temperature dependent, and delivered headspace concentrations can decline during treatment due to uptake by fruit and associated materials, increasing uncertainty when protocols are specified only by nominal dose and duration [14,22,23]. Moreover, quality impacts may not be fully expressed at treatment end and can emerge during subsequent refrigeration as acute stress responses translate into altered respiration patterns, cumulative mass loss, softening trajectories, and changes in decay incidence [24,25]. For cold-chain handling, where both insect response and berry physiology are temperature sensitive, the central question is whether EF can deliver reliable quarantine control while maintaining marketable blueberry quality during subsequent refrigerated storage, when commercial risk accumulates.

Although previous studies have shown that EF fumigation, alone or combined with cold treatment, can control *D. suzukii* in blueberries and other fruit systems, its translation into refrigerated blueberry export chains still requires a more integrated treatment framework [26,27,28]. Existing blueberry studies provide valuable evidence of EF efficacy, but temperature-specific treatment windows under cold-chain-relevant conditions remain insufficiently defined, particularly with respect to developmental-stage tolerance and the EF concentration required for reliable control. In addition, EF treatments are often described mainly by nominal concentration and exposure duration, whereas the actual headspace concentration during treatment is less frequently considered as an operational descriptor, despite its relevance to delivered exposure in fruit fumigation systems [29,30]. For highly perishable blueberries, phytosanitary efficacy also needs to be evaluated together with post-treatment quality during refrigerated storage, rather than only at treatment completion [17,19,24,25]. These gaps limit the translation of EF fumigation from a promising postharvest treatment into a practical cold-chain-compatible strategy that simultaneously satisfies quarantine efficacy, operational safety, and blueberry quality requirements. Therefore, the objective of this study was to define a cold-chain-compatible EF fumigation strategy for blueberries infested with *D. suzukii*. Specifically, this study aimed to identify the most tolerant developmental stage under low-temperature EF fumigation, quantify temperature-dependent concentration–mortality relationships for the target stage, monitor EF headspace dissipation under simulated refrigerated fumigation conditions, verify candidate schedules using zero-survivor tests under simulated cold-chain conditions, and evaluate post-treatment blueberry quality during refrigerated storage. By integrating stage-specific efficacy, realised fumigant exposure, verification-scale treatment performance, operational safety, and fruit quality preservation, this study provides new scientific evidence for implementing EF fumigation within refrigerated blueberry export logistics.

## 2. Materials and Methods

### 2.1. Fruit and Insects

The laboratory colony of spotted-wing drosophila (*Drosophila suzukii*) was provided by the Institute of Plant Protection, Shandong Academy of Agricultural Sciences (Shandong, China). The founding population was collected in 2024 from commercial cherry and grape orchards in Shandong Province. To maintain colony vigour and minimise laboratory adaptation, wild individuals were introduced approximately every 6 months. Insects were reared in insect cages under controlled-environment conditions at the Laboratory of Phytosanitary Treatment and Equipment Technologies, Chinese Academy of Quality and Inspection & Testing, at 25 ± 1 °C and 70 ± 5% relative humidity, with a 14 h light and 10 h dark photoperiod, and were maintained on an artificial diet composed of wheat bran, sucrose, and brewer’s yeast at a weight ratio of 8:4:1 [31]. Water, blueberries, and the adult diet were refreshed regularly to ensure continuous access to food and moisture.

Before fumigation bioassays, colony fitness was checked under standard rearing conditions by assessing fecundity, hatchability, pupation, adult emergence, adult longevity, and natural background survival. The colony showed high hatchability, pupation, adult emergence, and background survival, indicating stable baseline viability for the subsequent bioassays (Table S1).

Highbush blueberry fruit (*Vaccinium corymbosum*), cultivar ‘Brightwell’, produced in Yunnan Province, China, were purchased from a local fruit market. Fruit were selected to be free of visible disease symptoms, uniform in maturity and size, and without mechanical damage. Prior to processing, fruit were stored at 5 °C, and all experiments were completed within 7 days of arrival.

### 2.2. Developmental Test

To determine the developmental progression of *D. suzukii* in blueberry fruit, approximately 200 blueberries washed with sterile water were placed in a *D. suzukii* rearing cage for natural infestation and oviposition by adult flies. To obtain a relatively uniform infestation level, the oviposition exposure period was set to 6 h. After infestation, the blueberries were transferred to an environment-controlled chamber (KBF 720, BINDER GmbH, Tuttlingen, Germany) and maintained at 25 ± 1 °C and 60 ± 5% RH.

From day 1 to day 15 after infestation, infested blueberries were dissected every 24 h to observe and count the developmental stages of *D. suzukii* recovered from the fruit. To ensure accurate assignment of larval instars and developmental stages, body length, mouth-hook development, and body colour were used as morphological criteria, and recovered individuals were observed and measured under a stereomicroscope (SteREO Discovery-V12; Carl Zeiss, Jena, Germany) [14,15]. The proportion of each developmental stage was calculated as the number of individuals assigned to that stage divided by the total number of recovered individuals [32]. The experiment was conducted in three independent replicates.

### 2.3. Tolerance Test

Approximately 2 kg of blueberries were used for artificial inoculation with *D. suzukii* eggs, using a procedure modified from previous studies [33,34]. To ensure a broadly uniform infestation level among berries, each berry was inoculated with approximately 27 to 32 eggs. The inoculated blueberries were then maintained in an environment-controlled chamber (KBF 720, BINDER GmbH, Tuttlingen, Germany) at 25 ± 1 °C and 60 ± 5% relative humidity (RH) for 1, 2, 4, 7 and 11 days to obtain *D. suzukii* at the egg, 1st, 2nd, and 3rd instar larval, and pupal stages.

For fumigation, a 6 L desiccator (Leigu Technology Co., Ltd., Yancheng, China) containing 150 inoculated berries (loading factor approximately 5% (*w*/*v*)) was pre-cooled at 5 °C for 12 h in an environment-controlled chamber. The desiccator was then sealed using a modified rubber plug equipped with Swagelok snap fittings (304 stainless steel, 3/8 in., 8 mm thickness; Shanghai Yihao International Trading Co., Ltd., Shanghai, China) for gas-tight dosing. Fumigation was conducted in 6 L desiccators equipped with a small fan (DCF, model DD92DBVM-012, DC 12 V, 0.16 A, Guangzhou, China) and sealed using the same plug–fitting assembly to prevent gas leakage. Filter paper inserted into the rubber plug served as an evaporation substrate for liquid EF (reagent grade, 97% purity; Sigma-Aldrich, St. Louis, MO, USA), increasing the evaporation surface area and reducing local condensation during low-temperature fumigation. The EF dose required to achieve the target headspace concentration (30 mg·L^−1^) was calculated in advance, and a calculated injection volume of EF (0.2 mL) was injected into each desiccator using a gas-tight syringe (Hamilton Company, Reno, NV, USA) [14]. To maintain near-atmospheric pressure and avoid pressurization during dosing, an equivalent volume of headspace air was withdrawn immediately prior to EF injection. The fan was then switched on to promote rapid volatilization and homogeneous distribution of EF within the chamber. Separate fumigation trials were conducted at 5 °C, 10 °C, and 15 °C, each for 4 h at a target EF concentration of 30 mg·L^−1^.

EF concentrations were determined using a gas chromatograph equipped with a flame ionization detector (GC6890, Agilent Technologies Inc., Santa Clara, CA, USA; GC-FID). Separation was achieved using an HP-Innowax capillary column (30 m × 0.25 mm i.d., 0.25 μm film thickness; Agilent Technologies Inc., Santa Clara, CA, USA). High-purity nitrogen (99.99%) was used as the carrier gas at a flow rate of 0.02 mL·s^−1^ [35]. The oven temperature was set to 100 °C and held for 2.5 min, and the injector and detector temperatures were 200 °C and 250 °C, respectively.

After fumigation, the desiccators were opened for ventilation for 2 h, and the treated samples were then maintained under rearing conditions (25 ± 1 °C and 70 ± 5% RH) for different durations according to developmental stage. For each developmental stage, untreated control fruit were prepared from the same inoculation cohort and handled in parallel with the treated fruit, including the same grouping, transfer, and post-treatment holding procedures, while maintained under rearing conditions without EF dosing. For fruit containing eggs, treated blueberries were maintained for 7 days to allow surviving individuals to develop to later larval stages before survival assessment. For fruit containing 1st instar larvae, treated blueberries were also held for 7 days, and individuals that developed into 2nd or 3rd instar larvae were recorded as survivors. For fruit containing 2nd and 3rd instar larvae, treated blueberries were held for 3 days, and larvae that did not respond when prodded with a blunt probe were considered dead. For pupae, treated samples were maintained for 5 days to record adult emergence [32,33]. Mortality was calculated as the number of dead insects divided by the total number of insects assessed.

### 2.4. Toxicity Assay

Based on the tolerance comparison test, the most tolerant developmental stage of *D. suzukii* was identified and selected for further evaluation of its susceptibility to EF fumigation under the same experimental conditions described above. To assess the effect of temperature on EF toxicity, blueberry fruit artificially inoculated with known numbers of 1-day-old *D. suzukii* eggs were fumigated for 4 h at three temperatures (5, 10, and 15 °C) using a series of target EF concentrations (0, 30, 40, 50, 60, 70, and 80 mg·L^−1^). EF was introduced into the desiccators via surface evaporation. After confirming that EF was thoroughly mixed, headspace EF concentrations were monitored by gas chromatography (see Section 2.3). For each treatment, 150 blueberries containing approximately 4200 eggs in total (loading factor about 5%) were fumigated in 6 L desiccators at the target EF concentrations specified above. At the end of the exposure period, the desiccators were ventilated, and treated insects were transferred to rearing conditions for post-treatment development prior to mortality assessment. Each condition was replicated three times, with appropriate control groups included to account for background environmental effects.

### 2.5. Large-Scale Confirmatory Test

Based on the toxicity assay results, three candidate fumigation schedules were subjected to verification testing. For each treatment, approximately 3 kg of blueberries (1500 to 1800 berries) were continuously infested with *D. suzukii* in a rearing cage for 6 h and then maintained under rearing conditions for 24 h. A total of 150 untreated fruits were randomly selected from the same infested batch as controls and maintained under rearing conditions to allow egg development. The control fruit were then dissected, and the number of viable larvae recovered after development was used to estimate the infestation level per fruit.

The remaining infested fruit, together with an additional approximately 2 kg of healthy blueberries for quality evaluation (about 1000 to 1200 berries), were transferred into a 90 L stainless-steel fumigation chamber equipped with an internal fan (50 cm × 45 cm × 40 cm). The chamber was placed in an environment-controlled cabinet (KBF 720, BINDER GmbH, Tuttlingen, Germany) and pre-cooled at 5 °C for 12 h. The loading factor was approximately 5% (*w*/*v*), and all materials were evenly distributed within the chamber. Fumigation was initiated under negative pressure, with treatment temperatures set at 5 °C, 10 °C, and 15 °C to validate three candidate schedules derived from the toxicity assays (5 °C: 69 mg·L^−1^ EF; 10 °C: 83 mg·L^−1^ EF; 15 °C: 94 mg·L^−1^ EF). Prior to fumigant introduction, approximately 20% to 25% of the chamber air was evacuated. EF was then introduced stepwise in predetermined volumes using gas-tight syringes. Ambient air was subsequently released into the chamber to equilibrate pressure and initiate fumigation.

During fumigation, EF concentrations were measured at 10 min after dosing and at 1 h intervals thereafter. After fumigation, the chamber was vented in a fume hood for 2 h. The infested fruit were then maintained under rearing conditions and thoroughly dissected after 3 days. Any larvae showing movement or pupation were recorded as survivors. In addition, 150 untreated fruits were included at each treatment temperature and held under the same exposure duration (4 h) as controls to account for non-fumigation factors.

### 2.6. Quality Assessment of Blueberries

Based on the large-scale validation trials conducted at different temperatures, EF headspace concentrations were measured at 10 min, 1 h, 2 h, 3 h, and 4 h during fumigation. Concentration × time (Ct; mg·L^−1^·h) products were calculated using Equation (1) [36].

(1) $$Ct=\sum \left(C_{n}+C_{n+1}\right)\left(t_{n}+t_{n+1}\right)/2$$

where C is the fumigant concentration (mg·L^−1^), t is the exposure time (h), and n denotes the sampling order.

After fumigation, fruit were aerated in a fume hood for 2 h and then stored at 5 °C in a controlled-environment chamber. In phytosanitary studies of fresh horticultural commodities, 5 °C is commonly used as a low-temperature reference condition to improve comparability across studies. Blueberries were randomly sampled from the fumigated and control groups on Days 1, 7, and 14 for physicochemical quality measurements, and visible injury (decay and surface shrivelling or wilting) was recorded. Because temperature deviations can occur at multiple points in commercial cold chains (for example, phytosanitary inspection, transport, and packing), EF fumigation was conducted at 5, 10, and 15 °C to reflect a practical range of cold-chain temperature fluctuations and to support translation of the EF schedule into implementable, monitorable, and verifiable trade-compliance evidence.

Weight loss (WL) was calculated from the difference between the fruit weight measured at the end of each post-treatment storage period (W_t_) and the initial weight (W_0_). Fruit weight was determined using an analytical balance (Mettler Toledo, PL3001-S, Columbus, OH, USA). The calculation is shown in Equation (2) [37].

(2) $$Weight\;Loss\;\left(\%\right)=\frac{W_{0}-W_{t}}{W_{0}}\times 100$$

Blueberry firmness was measured using a texture analyser (TA-XT2i; Stable Micro Systems, Godalming, UK), with a force resolution of 0.001 N and a force accuracy of 0.025%. For measurement, each berry was positioned on the equatorial plane at the centre of the test platform and subjected to a puncture test using a 5 mm diameter cylindrical probe, with a penetration depth of 7 mm and a probe speed of 30 mm·min^−1^, ensuring that the probe fully penetrated the sample without contacting the platform surface. Firmness was obtained from the force–distance curve as the peak force, and mean values were calculated. For flesh firmness measurements, a thin layer of the epicarp was carefully removed prior to puncture testing.

After firmness measurement, blueberry fruits were homogenized and the juice was collected for the determination of total soluble solids (TSS) and titratable acidity (TA). TSS was measured using a handheld digital refractometer (GMK-701R, G-won Hitech, Seoul, Republic of Korea). For TA determination, the juice was diluted 100-fold and measured with an acidity meter (GMK-855, G-won Hitech). Both TSS and TA were measured in triplicate and recorded.

Blueberry respiration rate (RR) was assessed by monitoring CO_2_ accumulation in a sealed storage system. Each 6 L desiccator contained 35 blueberries (approximately 50 g), and three replicates were established. Gas samples (0.4 mL) were collected on days 1, 7, and 14 of storage. CO_2_ concentrations were measured by gas chromatography with a thermal conductivity detector (GC–TCD) using an 80/100-mesh Porapak Q packed column (Beijing Mingnike Analytical Instrument Equipment Center, Beijing, China). The column temperature was maintained at 70 °C, and hydrogen (H_2_) was used as the carrier gas at a flow rate of 0.3 mL·s^−1^. RR was expressed as mg CO_2_ kg^−1^·h^−1^ and calculated using Equation (3) [38].

(3) $$RR=\frac{\Delta C\times V\times 44}{M\times T\times 22.4}$$

where ΔC is the change in CO_2_ volume fraction (%), V is the desiccator volume (L), M is the fruit sample mass (kg), and T is the sealing time (h).

Sucrose and proanthocyanidin (PA) contents were determined using a Plant Sucrose Content Assay Kit (product no. BC2460; Solarbio, Beijing, China) and a Plant Proanthocyanidin Content Assay Kit (product no. BC1350; Solarbio, Beijing, China), respectively, based on visible spectrophotometric measurements. All assays were performed according to the manufacturers’ instructions.

The decay rate was expressed as the ratio (%) of decayed fruits relative to the total number of fruits. During cold storage, symptoms including peel injury, juice leakage, and microbial infection associated with chilling damage were recorded as decay [39].

### 2.7. Data Analysis

Observed mortality was corrected for control mortality using Abbott’s formula [40]. For the tolerance comparison, including low-temperature exposure alone and EF fumigation under low-temperature conditions, the control mortality was defined as the background mortality of insects maintained under laboratory rearing conditions at 25 ± 1 °C without EF treatment. For the toxicity assay, mortality at each EF concentration was corrected using the corresponding untreated control at the same treatment temperature (5 °C, 10 °C, or 15 °C). The means and standard errors (SEs) of replicates were calculated using Microsoft Excel (Microsoft 365 App for Enterprise). Mortality data were analysed in SPSS Statistics 27 (IBM Corp., Armonk, NY, USA) by analysis of variance, with mean separation conducted using Tukey’s honestly significant difference (HSD) test (*p* < 0.05). To compare EF tolerance of *D. suzukii* eggs among temperature conditions, mortality proportions were arcsine-transformed and dose–mortality relationships were evaluated using linear regression, with temperature effects tested by ANCOVA. Probit analysis (probit and logit model) was conducted using POLO PLUS software, version 2.0 (LeOra Software, Berkeley, CA, USA).

For zero-survivor verification tests, the confidence associated with treating n insects with no survivors was calculated using Equation (4) [33].

(4) $$1-Pu={\left(1-C\right)}^{1/n}$$

where *C* is the confidence level and *Pu* is the acceptable survival level (typically 0.01% or 0.0032%) [41].

Fruit quality attributes (WL, firmness, TSS, TA, sucrose content, RR, and PA content) were analysed in SPSS using two-way ANOVA, with Tukey’s HSD for mean comparisons (*p* < 0.05).

## 3. Results

### 3.1. Profiling the Developmental Biology of Drosophila suzukii

**Figure 2:**
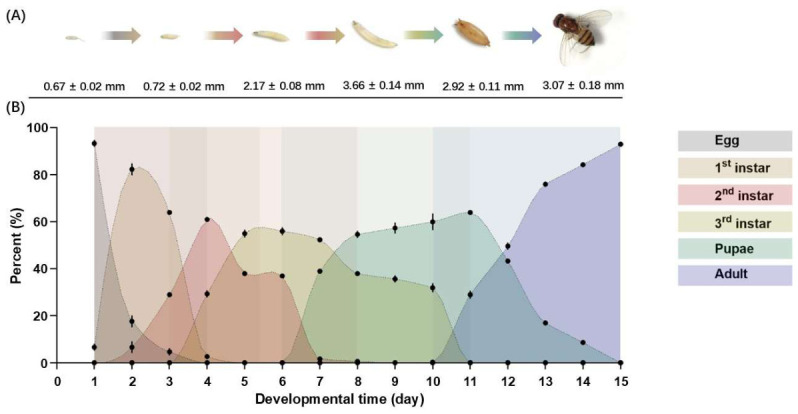
Developmental profile of *Drosophila suzukii* in infested blueberries, (**A**) stage-specific body length, (**B**) stage proportions over developmental time. Data are expressed as means ± SE (*n* = 3).

The development of *D. suzukii* comprises four stages (egg, larva, pupa, adult), with three larval instars (Figure 2A). Eggs are small, elongate-oval, and translucent to milky white. Larvae are cream-coloured, apodous maggots that are externally similar across instars, so instar identification relied on mouth-hook development together with body length ranges. Mean body length increased from eggs and early instars to the late-feeding 3rd instar (0.67 ± 0.02 mm, 0.72 ± 0.02 mm, 2.17 ± 0.08 mm, and 3.66 ± 0.14 mm for eggs, 1st, 2nd, and 3rd instars, respectively), then decreased in the more compact pupal stage (2.92 ± 0.11 mm) prior to adult emergence (3.07 ± 0.18 mm). Pupae formed a hardened, progressively pigmented puparium, and adults were readily distinguished by wings and fully developed adult morphology [42,43].

The proportional stage composition of *D. suzukii* recovered and counted from dissected blueberries showed a clear developmental progression over time (Figure 2B). Eggs represented the largest proportion on day 1 (about 93%) and declined as hatching began, with 1st instar larvae peaking on day 2 (about 82%) before second-instar larvae increased and represented the largest proportion by day 4 (about 60%). Third-instar larvae then represented the largest proportion from days 5 to 7 (about 52% to 56%), after which pupae increased rapidly and became dominant from days 8 to 11 (about 55% to 64%). Adult emergence accelerated after day 11, adults exceeded pupae by day 12 (about 48% versus 44%) and reached about 93% by day 15. High developmental success (egg hatch 92.5 ± 0.01%, pupal eclosion 99.6 ± 0.01%), together with high fecundity (19.38 ± 1.39 eggs per female per day) and adult longevity (16.77 ± 1.09 d), support strong establishment potential once oviposition occurs and provide a practical baseline for timing stage-targeted fumigation.

### 3.2. Life-Stage Differences in Fumigation Tolerance of Drosophila suzukii

Across the cold-chain-relevant temperature window of 5 to 15 °C, mortality responses differed markedly among developmental stages, and low-temperature effects were evident under both low-temperature exposure alone and EF fumigation under low-temperature conditions (Table 1a,b). Under low-temperature exposure alone, Abbott-corrected mortality increased as temperature decreased across all stages (Table 1a), and two-way ANOVA showed significant main effects of stage and temperature, with a significant stage × temperature interaction (stage: F(4,30) = 176.01; temperature: F(2,30) = 1835.20; stage × temperature: F(8,30) = 12.16; all *p* < 0.0001). Pupae exhibited the lowest mortality whereas eggs exhibited the highest, indicating stage-dependent sensitivity to low temperature [44].

Under EF fumigation under low-temperature conditions, corrected mortality for each developmental stage was substantially higher than the corresponding low-temperature–only Abbott-corrected mortality at the same temperature, indicating that EF remained the dominant lethal factor within the refrigerated handling window (Table 1b). Two-way ANOVA likewise showed strong main effects of stage and temperature, with a significant interaction (stage: F(4,30) = 561.07; temperature: F(2,30) = 153.79; stage × temperature: F(8,30) = 34.45; all *p* < 0.0001). Across temperatures, eggs were the most tolerant stage under EF fumigation, followed by pupae, whereas control of 1st and 2nd instar larvae was consistently high at all temperatures. Observed mortality of 3rd instar larvae was closer to that of pupae than to that of the early instars [23].

### 3.3. Toxicity Assay

#### 3.3.1. Linear Regression

To quantify egg tolerance across temperatures and test whether temperature alters the concentration–response slope, Abbott-corrected egg mortalities were arcsine-transformed and analysed by ANCOVA [29,45]. Both concentration and temperature were highly significant, and the concentration × temperature interaction was also significant (concentration: F(1,57) = 2632.95, *p* < 0.0001; temperature: F(2,57) = 91.44, *p* < 0.0001; concentration × temperature: F(2,57) = 6.43, *p* = 0.0030), indicating that the slope differed among temperatures rather than reflecting a simple parallel shift. Accordingly, separate linear regressions were fitted at each temperature to describe temperature-specific response functions (Table 2). These regressions showed excellent fit (R^2^ = 0.976 to 0.991) with slopes of 0.791 ± 0.019 (5 °C), 0.804 ± 0.031 (10 °C), and 0.853 ± 0.023 (15 °C), consistent with a modest temperature dependence in response steepness. Extrapolated “100% mortality” reference concentrations were 68.2, 74.9, and 82.9 mg·L^−1^ at 5, 10, and 15 °C, respectively.

#### 3.3.2. Probit Analysis

Across the three temperature regimes, the concentration to mortality relationships were well described by both link functions (Table 3a,b), with acceptable dispersion, as indicated by heterogeneity values close to 1. Under the probit model, lethal concentration estimates increased with temperature, with LC_50_ rising from 26.756 mg·L^−1^ at 5 °C (95% CL 23.997 to 29.029) to 31.358 mg·L^−1^ at 10 °C (28.067 to 34.084) and 36.880 mg·L^−1^ at 15 °C (34.449 to 39.019), and the same monotonic increase was observed for LC_99_ (69.061, 83.863, and 93.644 mg·L^−1^, respectively) and the probit 9 endpoint LC_99.9968_ (136.627, 170.198, and 183.084 mg·L^−1^). The logit model yielded very similar mid-range potency estimates, with LC_50_ values of 27.335, 31.469, and 36.888 mg·L^−1^ at 5, 10, and 15 °C, respectively, indicating that inference around the central part of the response curve was robust to the choice of link. In contrast, model dependence became pronounced at the extreme tail that is most relevant to phytosanitary assurance, because extrapolation to near complete control amplifies small differences in the fitted slope and link function. Consistent with this, logit based LC_99.9968_ estimates were substantially higher and less precise than probit based values at all temperatures, reaching 264.777, 385.805, and 423.697 mg·L^−1^ at 5, 10, and 15 °C, respectively, with markedly wider confidence limits. Although probit and logit provided similarly good fits across the observed mortality range, extrapolation to near-complete control was highly model-dependent, with probit-9 equivalents diverging markedly between link functions, reflecting uncertainty in the extreme tail rather than differences supported by the bioassay data. In addition to this statistical sensitivity, EF schedule nomination is constrained by flammability limits in air (LEL 2.94% *v*/*v*; UEL 16.84% *v*/*v*), which correspond to approximately 92.1 to 95.4 mg·L^−1^ (LEL) and 527.6 to 546.6 mg·L^−1^ (UEL) at 1 atm over 5 to 15 °C; notably, the probit-derived LC_99_ values (69.061 to 93.644 mg·L^−1^) remain below the LEL, whereas LC_99.9968_ (136.627 to 183.084 mg·L^−1^) exceeds it, reinforcing LC_99_-class nomination as the practical upper bound for safe implementation [46,47].

### 3.4. Ethyl Formate Sorption Dynamics in Blueberries

**Figure 3:**
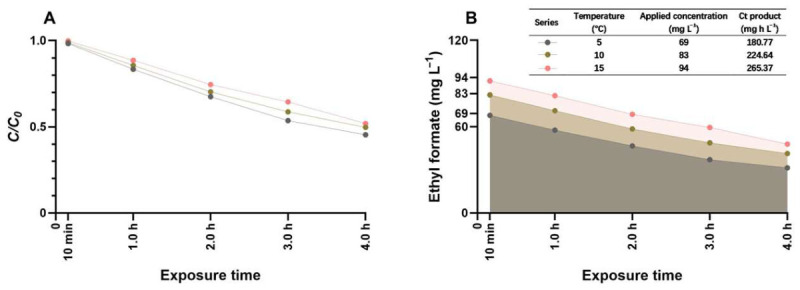
(**A**) Sorption-driven depletion of ethyl formate from the headspace, plotted as the ratio (C/C_0_) of measured concentration (C) to the applied initial concentration (C_0_) against time, values are means across sampling time points. (**B**) Temperature-dependent EF headspace concentration decay and the resulting concentration × time (Ct) exposure, at 5, 10, and 15 °C.

Under sealed 90 L fumigation conditions with a low loading factor of approximately 5% (*w*/*v*), EF exhibited a consistent concentration profile at 5, 10, and 15 °C, characterised by rapid establishment of the headspace concentration followed by progressive depletion over the exposure period (Figure 3A,B). Across all three temperatures, the normalised concentration remained close to unity at 10 min (C/C_0_ = 0.983, 0.989, and 0.998), but then declined steadily to 0.835, 0.857, and 0.886 at 1 h, 0.675, 0.667, and 0.745 at 2 h, 0.537, 0.587, and 0.645 at 3 h, and 0.454, 0.497, and 0.519 at 4 h (Figure 3A), indicating a sustained sorptive sink associated with the commodity and chamber matrices [29,48]. Despite broadly similar proportional depletion across temperatures, cumulative exposure increased with temperature, with Ct products of 180.77, 224.64, and 265.37 mg·h·L^−1^ at 5, 10, and 15 °C, respectively (Figure 3B). This trend primarily reflects differences in applied setpoints and the overall elevation of the concentration–time profile, rather than a qualitative change in the depletion mechanism.

### 3.5. Large-Scale Confirmatory Trials

Large-scale confirmatory trials translated the candidate EF treatment intensities identified from the dose–response modelling into auditable evidence of phytosanitary security and verified their efficacy under low-temperature conditions (Table 4). Based on the number of viable larvae recovered from untreated control fruit after development, the infestation level of *D. suzukii* eggs was estimated at approximately 29 to 32 eggs per berry. Based on this infestation rate, an estimated total of 31,164, 32,312, and 33,047 *D. suzukii* eggs were treated under each treatment condition. At 5, 10, and 15 °C, the selected EF concentrations delivered complete control of the most tolerant stage, 1-day-old *D. suzukii* eggs, and achieved phytosanitary treatment efficacies of 99.9904% to 99.9909% at the 95% confidence level, meeting the minimum evidentiary requirement for phytosanitary treatment validation in international trade [33,49].

### 3.6. Fruit Quality Evaluation

#### 3.6.1. Physicochemical Quality Attributes

**Figure 4:**
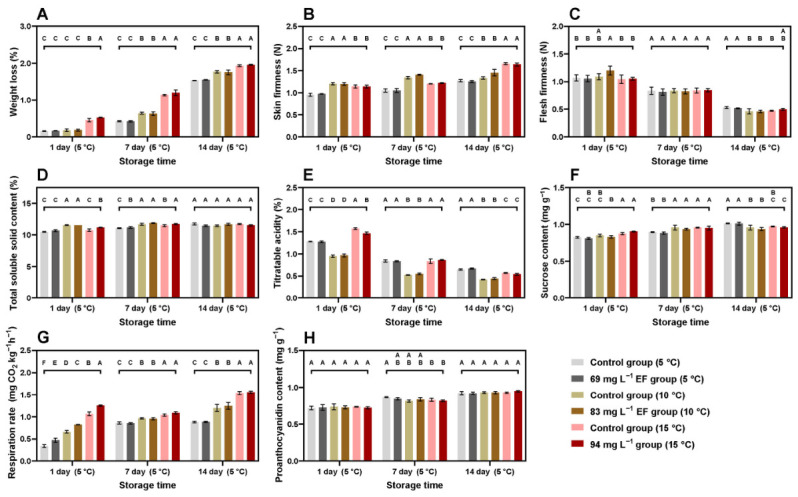
Changes in blueberry quality attributes during storage following ethyl formate fumigation conducted at 5, 10, and 15 °C, including weight loss (**A**), skin firmness (**B**), flesh firmness (**C**), total soluble solids (**D**), titratable acidity (**E**), sucrose content (**F**), respiration rate (**G**), and proanthocyanidin content (**H**), evaluated after 1 day, 7 days, and 14 days of storage. Data are presented as mean ± SE. Different letters indicate significant differences based on Tukey’s HSD test at *p* < 0.05.

Across the cold-chain-relevant temperature window of 5 to 15 °C, blueberry quality dynamics were driven primarily by the combined effects of storage time and temperature, whereas the EF treatment group exerted only minor influences on most attributes and these were largely confined to early or context-dependent responses (Figure 4A–H). Overall, progressive storage led to marked increases in WL, declines in flesh firmness, decreases in TA, and increases in RR, consistent with typical blueberry postharvest physiology in which moisture loss and cell-wall disassembly underpin textural softening and continued metabolism consumes organic acids, with higher temperatures accelerating both water migration and respiratory turnover [19,50]. WL showed significant temperature effects at 1 day, 7 days, and 14 days, indicating that warming substantially accelerated cumulative water loss; a treatment effect was detected only at 1 day (treatment, F(1,12) = 5.59, *p* = 0.0358) with no significant interaction (F(2,12) = 3.33, *p* = 0.0707), suggesting that EF-associated differences in WL were transient rather than persistent [19,51]. Texture changes in skin and flesh firmness were driven mainly by storage temperature rather than EF treatment. Skin firmness showed strong temperature effects at 1, 7, and 14 days (1 day: F(2,18) = 12.85, *p* = 0.0004; 7 days: F(2,18) = 26.40, *p* < 0.0001; 14 days: F(2,18) = 106.61, *p* < 0.0001), with no consistent main effect of treatment. Flesh firmness likewise exhibited significant temperature effects at 1 and 14 days (1 day: F(2,18) = 10.20, *p* = 0.0011; 14 days: F(2,18) = 16.44, *p* < 0.0001), whereas treatment and temperature × treatment terms were not significant overall, indicating that firmness changes were governed primarily by storage conditions rather than EF-related injury. Consistently, within-time-point letter groupings separated treatments mainly along the temperature gradient, with minimal divergence between the control and EF groups within the same temperature (Figure 4A–C) [14,19]. In contrast to the strong time and temperature dependence of physical attributes, TSS and sucrose remained within relatively narrow ranges, implying broadly stable sugars under cold-chain storage and suggesting that “flavor drift” was more strongly associated with acidity changes; TSS displayed significant interactions at 1 day and 14 days (1 day: temperature × treatment, F(2,12) = 6.39, *p* = 0.0129; 14 days: F(2,12) = 6.38, *p* = 0.0129), while main effects at 14 days were not significant (temperature, F(2,12) = 0.21, *p* = 0.8167; treatment, F(1,12) = 1.88, *p* = 0.1952), indicating that late-stage differences could be detected as a combined pattern but were not large or consistent enough to yield stable separation among groups. TA exhibited a stronger temperature dependence and also showed interactions at 1 day and 14 days (1 day: F(2,12) = 11.59, *p* = 0.0016; 14 days: F(2,12) = 6.18, *p* = 0.0143), implying that the EF treatment group did not uniformly increase or decrease acidity but instead modified acidity in a temperature-contingent manner, plausibly reflecting temperature-regulated metabolic rate and organic-acid turnover [52]. RR was consistently dominated by temperature effects across all time points, and the EF treatment group showed a treatment effect only at 1 day (treatment, F(1,12) = 117.15, *p* < 0.0001), with treatment and interaction effects not significant at 7 days and 14 days, indicating that EF-related perturbation to respiration was primarily an early response that was subsequently overridden by storage temperature and time; this aligns with Figure 4G, where the control group and the EF treatment group at the same temperature often share the same significance grouping at later storage times [53]. PA content fluctuated only slightly, aligning with a profile in which storage conditions drive gradual drift rather than treatment-induced discontinuities and supporting the view that blueberry phenolics are comparatively stable under refrigerated conditions, with more pronounced shifts typically emerging under warmer or extended storage scenarios (Figure 4H) [54].

#### 3.6.2. External Appearance Quality During Storage

**Figure 5:**
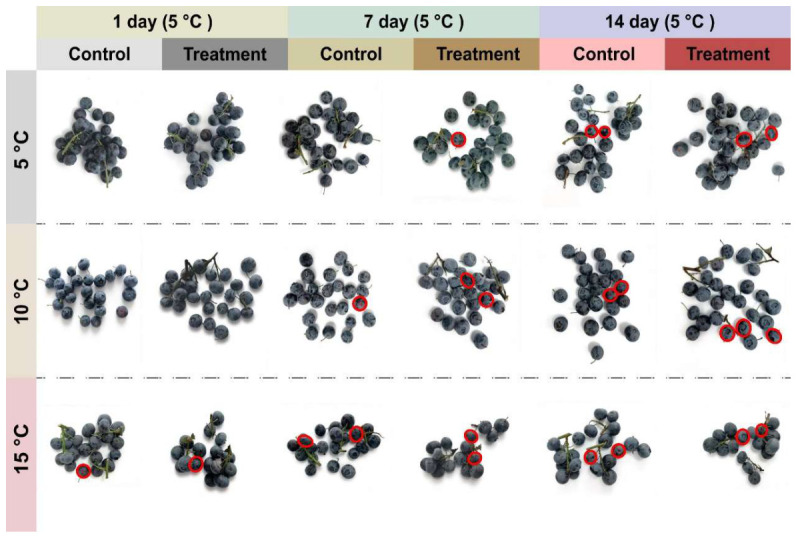
Appearance changes in blueberries during storage after ethyl formate fumigation at 5, 10, and 15 °C. Representative surface appearance of the control group and the EF treatment group is shown after 1, 7, and 14 days of storage. Red circles highlight individual berries exhibiting visible deterioration symptoms, including slight shrivelling, wilting, soft patches, or local depressions.

Across all temperature-by-treatment combinations, decay incidence remained zero, indicating that EF, at the treatment intensity and storage duration tested, did not induce a detectable increase in decay risk. In contrast to decay, the proportion of shrivelled or wilted berries accumulated progressively with storage time, and the soft patches, local depressions, and shrivelling highlighted by the red circles in Figure 5 are consistent with appearance deterioration dominated by moisture loss. This pattern accords with the well-recognised sensitivity of blueberries to time–temperature history and their propensity for water loss–associated declines in visual marketability during storage [55]. Critically, within the same temperature and storage time, the EF treatment group did not show a stable or consistent separation from the control group, providing no evidence of a persistent EF-related effect on appearance. Accordingly, the modest fluctuations observed at individual time points are more plausibly attributable to biological variability among fruit and natural variation in moisture-loss progression, rather than systematic EF-induced appearance injury (Table 5) [26]. Overall, these findings provide direct evidence that EF fumigation can be implemented under cold-chain-relevant low-temperature conditions while maintaining acceptable appearance during subsequent refrigerated storage, aligning with the study objective of supporting transferable quality-safety assurance for phytosanitary compliance without imposing an apparent appearance cost.

## 4. Discussion

The present study showed that the efficacy of EF against *D. suzukii* in blueberries was strongly shaped by both life stage and treatment temperature. Eggs remained the most tolerant stage under EF fumigation across 5 to 15 °C, whereas low-temperature exposure alone produced a different stage-response pattern. This indicates that the limiting stage in the present system was determined more by the biological mode of stress imposed by EF than by cold exposure itself. This result is consistent with the blueberry study of Kwon et al., who also identified eggs as the most tolerant stage of *D. suzukii* to EF and reported that EF was less effective at 5 °C than at 21 °C, with higher LCt values required under the lower temperature condition [22,26,44]. In that respect, the present study agrees with earlier evidence that EF efficacy against *D. suzukii* declines as treatment temperature decreases, even though the treatment remains operationally useful under cold-chain conditions.

A plausible explanation for this temperature effect is that EF toxicity depends not only on external concentration, but also on the rate at which a biologically effective internal dose is formed and expressed. Earlier toxicological work on alkyl formates showed that EF is rapidly metabolised to formic acid in insects and that formate toxicity is closely associated with inhibition of cytochrome C oxidase in the mitochondrial electron transport chain [46,56,57]. This mode of action links EF lethality to respiratory metabolism rather than to simple contact injury alone. Under lower temperatures, insect metabolism and respiratory flux are suppressed, which may slow the uptake, activation, or toxic expression of EF and thereby reduce acute fumigant efficacy. This interpretation is further supported by metabolomic work in *D. suzukii*, which showed that combined ethyl formate and low-temperature treatment altered pathways associated with the tricarboxylic acid cycle, nucleotide metabolism, glutathione metabolism, and redox balance [58].

The stage-specific pattern observed here is also biologically consistent with previous studies. Eggs are likely to be less responsive to EF because they are structurally protected and developmentally less metabolically active than feeding larval stages. By contrast, 1st and 2nd instar larvae were highly susceptible across temperatures in the present study, which is in line with the view that actively feeding and respiring stages are more vulnerable to fumigants targeting respiratory metabolism. Kwon et al. (2021) similarly found that eggs were the most tolerant stage of *D. suzukii* to ethyl formate in blueberries, while Jeon et al. (2022) showed that stage-specific responses remained important when ethyl formate was combined with cold treatment [22,39]. More broadly, the biological importance of egg protection and internal infestation in *D. suzukii* fruit systems has been emphasized in foundational work on this pest’s oviposition behavior and invasion biology [7,42]. Taken together, these studies support the interpretation that eggs represent the restrictive stage for ethyl formate-based phytosanitary treatment of *D. suzukii* in fruit systems. The shift in stage-specific tolerance ranking between low-temperature exposure alone and EF fumigation suggests that EF-driven toxicity, rather than chilling stress alone, was the dominant factor determining stage responses under refrigerated fumigation conditions [25,27]. EF-induced mortality is likely governed by the formation of a biologically effective internal dose, which may be limited by gas exchange, diffusion barriers, and metabolic activity in structurally protected or lower-metabolic stages such as eggs and pupae [56,59]. From an implementation perspective, eggs are a more restrictive target than later larvae because they remain embedded within apparently healthy fruit and are difficult to detect by visual inspection. Although 3rd instar larvae may show relatively high tolerance in some contexts, mature larvae can leave fruit and may be more readily reduced during grading, washing, or handling [7,10]. Therefore, dose selection and confirmatory validation should be anchored to cryptic and relatively tolerant stages, particularly eggs, when developing cold-chain-compatible EF protocols for *D. suzukii* in blueberries [60].

The present results also extend previous work by showing that this stage-specific pattern remains stable across a refrigerated 5 to 15 °C window and by quantifying how treatment concentration must be adjusted with temperature. This is an important practical point. Earlier studies demonstrated that EF could control *D. suzukii* in blueberries at low temperature and that low-dose EF could complement cold treatment, but they did not fully resolve the temperature-dependent concentration-response relationship across multiple cold-chain-relevant conditions [22,44]. The current study therefore strengthens the evidence base by showing that egg tolerance increases progressively from 5 to 15 °C and that treatment schedules should be temperature-specific rather than transferred unchanged across cold-chain scenarios [61,62].

Another important outcome was the marked divergence between model estimates at the extreme high-mortality tail. Probit and logit models produced similar LC_50_ values, but LC_99.9968_ estimates differed substantially, indicating that inference near complete mortality was highly model-dependent. For this reason, the present study did not rely solely on extreme model-derived endpoints for treatment nomination. Instead, candidate concentrations were selected from the dose–response analysis and then validated by confirmatory zero-survivor trials. This approach was particularly important for EF because schedule nomination is also constrained by flammability limits in air [45,50]. In the present study, the probit-derived LC_99_ estimates remained within the practical safety boundary, whereas LC_99.9968_ estimates exceeded the lower flammability limit. Therefore, LC_99_-class concentrations, followed by confirmatory zero-survivor validation, provided a more operationally realistic and defensible basis for schedule development than direct adoption of extreme extrapolated endpoints alone [50]. This provides a stronger phytosanitary basis for schedule development than tail extrapolation alone, particularly for EF, where treatment intensity is also limited by flammability constraints [26,46].

The sorption results support this treatment-development logic. EF concentration declined progressively from the headspace during the 4 h exposure period at all temperatures, indicating substantial depletion during fumigation. Similar patterns have been reported in other EF commodity systems and are generally attributed to sorption by the commodity and chamber matrices [30]. This means that nominal concentration and exposure duration do not fully describe realised treatment intensity. Accordingly, Ct and concentration–time dynamics are more informative than nominal dose alone when comparing treatment performance or translating schedules to larger systems [22,26].

The confirmatory trials further demonstrated the operational relevance of the selected schedules. At all three temperatures, the nominated EF concentrations achieved complete control of the most tolerant stage under simulated cold-chain conditions. This is relevant because commercial blueberry cold chains may experience short-term temperature fluctuations during precooling, grading, packing, loading, unloading, or inspection [63]. Under such conditions, refrigeration may suppress insect development but does not necessarily provide auditable phytosanitary security, and pest physiology may shift from cold-induced suppression toward metabolic recovery [58,63]. Therefore, a treatment that remains effective across a 5 to 15 °C window provides a more practical basis for managing risk during cold-chain handling. These results indicate that efficacy observed in analytical-scale bioassays remained robust at confirmatory scale. At the same time, most measured fruit-quality attributes were influenced mainly by storage time and temperature rather than by EF treatment. The main EF-associated response was a transient increase in respiration early in storage, but this did not lead to persistent deterioration in the measured physicochemical or visual traits during the storage period. Visible deterioration was generally mild and occurred only in a small proportion of berries, mainly as slight shrivelling, wilting, soft patches, or local depressions. This pattern is consistent with moisture-loss-associated appearance deterioration during storage rather than a stable EF-induced injury response [55]. Together, these findings suggest that the selected EF schedules were compatible with refrigerated blueberry storage under the conditions tested [14,19,24].

Overall, the present findings are broadly consistent with previous studies in showing that EF can be an effective postharvest option against *D. suzukii* in blueberries and that eggs are the key restrictive stage. At the same time, this study adds two important points to the existing literature: first, it shows more clearly that EF performance within a refrigerated handling window is temperature-dependent and must be interpreted through both toxicological response and gas-delivery dynamics; second, it demonstrates that schedules selected within practical safety limits can still achieve complete control at confirmatory scale without causing clear deterioration of the measured blueberry quality traits during storage. In that sense, the study does not merely repeat earlier findings, but refines them into a more operationally relevant framework for cold-chain phytosanitary treatment.

## 5. Conclusions

This study demonstrates that EF can deliver quarantine-grade disinfestation of *Drosophila suzukii* in blueberries within a cold-chain compatible 5 to 15 °C window, supporting EF as a greener, residue-free alternative to legacy fumigants. Efficacy was strongly stage dependent, with 1-day-old eggs identified as the most tolerant stage, and temperature-specific concentration–response relationships under a 4 h exposure supported nomination of candidate schedules. These schedules were translated from model-based targets to auditable evidence through verification-scale zero-survivor testing, providing an operational basis for phytosanitary implementation under refrigerated handling. Importantly, schedule setting was explicitly bounded by EF flammability constraints in air, and the selected treatment intensities were nominated to remain within the flammability safety boundary while maintaining reliable control across the tested temperature range.

From a food quality and shelf-life perspective, the nominated cold-chain schedules did not compromise blueberry marketability during subsequent refrigerated storage. Quality changes were driven primarily by storage time and temperature, while EF introduced no detectable or persistent penalties in external appearance or decay development. Across 1, 7, and 14 days of cold storage, weight loss and firmness followed expected storage-related trajectories, whereas key market-relevant physicochemical attributes, including total soluble solids, titratable acidity, sucrose, and proanthocyanidins, were not adversely affected by EF exposure. EF effects were mainly expressed as a transient elevation in respiration early in storage, without translating into cumulative quality loss. Collectively, these findings indicate that phytosanitary-level control can be achieved within refrigerated logistics while preserving the quality attributes that underpin consumer acceptance and commercial value.

Future work should prioritise commercial translation by validating treatment delivery and Ct uniformity under realistic loading rates and packaging formats, testing robustness under typical cold-chain temperature fluctuations and handling interruptions, and extending post-treatment quality tracking across longer distribution and retail shelf-life durations.

## Figures and Tables

**Table 1:** (**a**) Abbott-corrected mortality (%) of *Drosophila suzukii* across developmental stages under low-temperature exposure alone, corrected relative to the 25 ± 1 °C laboratory-rearing control. (**b**) Abbott-corrected mortality (%) of *Drosophila suzukii* across developmental stages after ethyl formate fumigation under low-temperature conditions, corrected relative to the 25 ± 1 °C laboratory-rearing control.

(**a**)				
**Developmental Stages**	**Temperature-Matched Corrected Mortality (%)**			**Means of Developmental Stages (%)**
	**15 °C**	**10 °C**	**5 °C**	
1 day-old eggs	12.52 ± 0.70 cA	21.62 ± 0.70 bA	28.62 ± 0.00 aA	20.92 ± 4.66 A
1st instar larvae	8.32 ± 0.35 cB	19.52 ± 0.35 bA	26.67 ± 0.16 aB	18.17 ± 5.34 A
2nd instar larvae	7.97 ± 0.35 cB	17.42 ± 0.70 bB	24.85 ± 0.19 aC	16.75 ± 4.88 A
3rd instar larvae	6.22 ± 0.35 cC	14.97 ± 0.73 bB	24.11 ± 0.19 aC	15.10 ± 5.16 A
Pupae	3.77 ± 0.35 cD	8.44 ± 0.42 bC	22.32 ± 0.51 aD	11.51 ± 5.57 A
(**b**)				
**Developmental Stages**	**Corrected Mortality of Fumigated *D. suzukii* (%)**			**Means of Developmental Stages (%)**
	**15 °C**	**10 °C**	**5 °C**	
1 day-old eggs	44.71 ± 0.70 cD	58.71 ± 0.70 bD	76.21 ± 0.70 aC	59.88 ± 9.11 C
1st instar larvae	98.60 ± 1.40 aA	98.60 ± 1.40 aA	97.67 ± 2.33 aA	98.29 ± 0.31 A
2nd instar larvae	97.90 ± 1.21 aA	97.90 ± 1.21 aA	97.40 ± 0.27 aA	97.73 ± 0.17 A
3rd instar larvae	67.98 ± 1.09 cB	74.92 ± 0.31 bB	84.84 ± 2.33 aB	75.91 ± 4.89 B
Pupae	61.16 ± 1.21 bC	63.96 ± 0.93 bC	82.93 ± 0.91 aB	69.35 ± 6.84 BC

Note: Corrected mortality (%) was calculated using Abbott’s formula with control mortality. Values are mean ± SE. Different lower-case letters within rows and upper-case letters within columns indicate significant differences (Tukey’s HSD test, *p* < 0.05). Different letters in the final column indicate significant differences in mean mortality of developmental stages under low-temperature treatment alone (a) and after EF fumigation under low-temperature conditions (b) (Tukey’s HSD test, *p* < 0.05).

**Table 2:** Linear regressions on concentration–mortality data for *Drosophila suzukii* eggs under ethyl formate fumigation at 5, 10, and 15 °C.

Temperature	Observations	y-Intercept (Mean ± SE)	Slope (Mean ± SE)	R^2^	Predicted Dose for 100% Mortality (mg·L^−1^)
5 °C	18	36.05 ± 0.91	0.791 ± 0.019	0.991	68.2
10 °C	18	29.78 ± 1.48	0.804 ± 0.031	0.976	74.9
15 °C	21	19.29 ± 1.22	0.853 ± 0.023	0.986	82.9

**Table 3:** (**a**) Probit-derived lethal concentration estimates for ethyl formate fumigation against 1-day-old eggs of *Drosophila suzukii* at 5, 10, and 15 °C with a 4 h exposure. (**b**) Logit-derived lethal concentration estimates for ethyl formate fumigation against 1-day-old eggs of *Drosophila suzukii* at 5, 10, and 15 °C with a 4 h exposure.

(**a**)								
**Temperature**	**No. of Insects**	**Slope ± SE**	**Chi-Square**	**df ^a^**	**Heterogeneity**	**Probit Analysis of Lethal Concentration (mg·L^−1^) (95% CL ^b^)**		
						**LC_50_**	**LC_99_**	**LC_99.9968_**
5 °C	76,092	5.649 ± 0.499	8.686	16	0.543	26.756 (23.997–29.029)	69.061 (63.166–77.904)	136.627 (113.903–176.361)
10 °C	75,188	5.445 ± 0.397	22.716	16	1.420	31.358 (28.067–34.084)	83.863 (75.280–97.394)	170.198 (137.980–230.026)
15 °C	76,114	5.749 ± 0.342	19.713	16	1.232	36.880 (34.449–39.019)	93.644 (85.665–105.004)	183.084 (155.073–227.755)
(**b**)								
**Temperature**	**No. of Insects**	**Slope ± SE**	**Chi-Square**	**df**	**Heterogeneity**	**Logit Analysis of Lethal Concentration (mg·L^−1^) (95% CL)**		
						**LC_50_**	**LC_99_**	**LC_99.9968_**
5 °C	76,092	10.506 ± 0.948	12.440	16	0.777	27.335 (24.872–29.373)	74.833 (67.015–87.190)	264.777 (197.562–401.717)
10 °C	75,188	9.518 ± 0.718	26.349	16	1.647	31.469 (28.094–34.248)	95.639 (82.513–118.779)	385.805 (264.779–681.010)
15 °C	76,114	9.773 ± 0.620	24.510	16	1.532	36.888 (34.145–39.268)	108.915 (95.877–129.665)	423.697 (309.163–657.476)

^a^ Degrees of freedom; ^b^ Confidence limits.

**Table 4:** Large-scale confirmatory testing of candidate ethyl formate concentrations at 5, 10, and 15 °C for quarantine disinfestation against 1-day-old eggs of *Drosophila suzukii* in blueberries.

Temperature	EF Concentration	No. of Test Fruit	No. of Eggs	Estimate Total Number of Treated Insects ^a^	Mortality	Estimated Efficacy
5 °C	69 mg·L^−1^	1050	0	31,164	100%	99.9904%
	Control	150	4452	-	-	-
10 °C	83 mg·L^−1^	1050	0	32,312	100%	99.9907%
	Control	150	4616	-	-	-
15 °C	94 mg·L^−1^	1050	0	33,047	100%	99.9909%
	Control	150	4721	-	-	-

^a^ The total numbers of treated insects were calculated from the infestation rate of their control fruit and the number of treated fruit.

**Table 5:** Changes in blueberry fruit appearance (percentage of shrivelled/wilted berries) after 1, 7, and 14 days of cold storage (%).

Temperature	Treatment	No. of Test Fruit	1 Day (%)	7 Day (%)	14 Day (%)
5 °C	69 mg·L^−1^	150	0.00 ± 0.00 bA	1.33 ± 1.15 bA	3.33 ± 1.15 aA
	Control	150	0.00 ± 0.00 bA	0.67 ± 1.15 bA	2.67 ± 1.15 aA
10 °C	83 mg·L^−1^	150	0.00 ± 0.00 bA	3.33 ± 1.15 abA	4.00 ± 2.00 aA
	Control	150	0.00 ± 0.00 bA	1.33 ± 1.15 bA	3.33 ± 1.15 aA
15 °C	94 mg·L^−1^	150	1.33 ± 1.15 bA	4.00 ± 2.00 abA	5.33 ± 1.15 aA
	Control	150	0.67 ± 1.15 bA	3.33 ± 1.15 abA	4.67 ± 1.15 aA

Note: Values are mean ± SE (%). Different lower-case letters within each row indicate significant differences among storage times, and different upper-case letters within each column indicate significant differences among treatments (Tukey’s HSD test, *p* < 0.05).

## Data Availability

The original contributions presented in this study are included in the article/Supplementary Materials. Further inquiries can be directed to the corresponding author.

## Supplementary Materials

The following supporting information can be downloaded at: https://www.mdpi.com/article/10.3390/insects17060580/s1. Table S1: Background colony performance parameters of *Drosophila suzukii* under laboratory rearing conditions.
