# Supplementary material for: Cold-Chain Compatible Ethyl Formate Fumigation for Phytosanitary Disinfestation of Drosophila suzukii in Blueberries
Source: Insects. 2026 Jun 2;17(6):580. doi: 10.3390/insects17060580 (PMC13301318; doi:10.3390/insects17060580)
Supplement: Supplementary file 1 [file insects-17-00580-s001.zip › insects-4285519-supplementary.pdf]

Supplementary: Laboratory colony quality control of *Drosophila suzukii*

Table S1. Background colony performance parameters of *Drosophila suzukii* under laboratory rearing conditions.

Colony performance parameters	n	Mean ± SE	Range
Fecundity (eggs female^{-1}·d^{-1})	47	20.04 ± 1.93	17-24
Hatchability (%)	300	95.88 ± 2.59	91-99
Pupation (%)	300	95.40 ± 0.02	95-96
Adult emergence (%)	300	99.36 ± 1.15	96-100
Adult longevity (d)	43	16.82 ± 0.93	15-19
Natural (background) survival (%)	300	95.26 ± 1.97	92-100

Note: Values are mean ± SE. n denotes the total number of observations. Control mortality refers to background mortality under laboratory rearing conditions.
